# Motor-Skill Learning in an Insect Inspired Neuro-Computational Control System

**DOI:** 10.3389/fnbot.2017.00012

**Published:** 2017-03-08

**Authors:** Eleonora Arena, Paolo Arena, Roland Strauss, Luca Patané

**Affiliations:** ^1^Dipartimento di Ingegneria Elettrica, Elettronica, e Informatica, University of CataniaCatania, Italy; ^2^National Institute of Biostructures and BiosystemsRome, Italy; ^3^Institut für Zoologie III (Neurobiologie), University of MainzMainz, Germany

**Keywords:** insect brain, insect mushroom bodies, spiking neural controllers, learning, goal-oriented behavior

## Abstract

In nature, insects show impressive adaptation and learning capabilities. The proposed computational model takes inspiration from specific structures of the insect brain: after proposing key hypotheses on the direct involvement of the mushroom bodies (MBs) and on their neural organization, we developed a new architecture for motor learning to be applied in insect-like walking robots. The proposed model is a nonlinear control system based on spiking neurons. MBs are modeled as a nonlinear recurrent spiking neural network (SNN) with novel characteristics, able to memorize time evolutions of key parameters of the neural motor controller, so that existing motor primitives can be improved. The adopted control scheme enables the structure to efficiently cope with goal-oriented behavioral motor tasks. Here, a six-legged structure, showing a steady-state exponentially stable locomotion pattern, is exposed to the need of learning new motor skills: moving through the environment, the structure is able to modulate motor commands and implements an obstacle climbing procedure. Experimental results on a simulated hexapod robot are reported; they are obtained in a dynamic simulation environment and the robot mimicks the structures of *Drosophila melanogaster*.

## 1. Introduction

Recent results and experiments performed on insects shed light on their highly developed learning and proto-cognitive capabilities enabling them to adapt extremely well to their natural environment (Menzel and Giurfa, 1996; Liu et al., 1999; Tang and Guo, 2001; Chittka and Niven, 2009). Modeling insect brains is an increasingly important issue for the design of learning and control strategies to be applied on autonomously walking robots. Within the insect brain an important paired neuropil with higher control functions are the mushroom bodies (MBs), recently used to model different behavioral functions (Smith et al., 2008; Arena et al., 2013c). Studies on bees and flies identified the MBs as a relevant area for associative learning and memory in odor conditioning experiments (Menzel and Muller, 1996; Menzel, 2001; Scherer et al., 2003; Liu and Davis, 2006). MBs are also involved in behaviors depending on other sensory modalities, like vision (Liu et al., 1999; Menzel, 2001; Tang and Guo, 2001), other types of learning such as choice behaviors (Tang and Guo, 2001; Gronenberg and Lopez-Riquelme, 2004; Brembs, 2009) and, as recently introduced, also in the improvement of gap-climbing tasks (Pick and Strauss, 2005; Kienitz, 2010).

MBs receive olfactory input from the antennal lobes via projection neurons. The latter run in the medial antennal lobe tract, provide input to the MB calyces and continue on to the lateral horn (LH). The mediolateral and the lateral antennal lobe tracts emerge from the antennal lobes as well, but bypass the calyces and project directly to the LH. The LH region controls inborn behavior, whereas the MBs are thought to be involved in learnt behavior. Analysing the interaction between the different neural structures we investigated the emergence of interesting neural activities responsible for specific behaviors in insects, including flies, like attention, expectation, delayed-match to sample tasks, and others (Arena et al., 2012a,b, 2013b).

Major dynamical aspects characterizing the locust olfactory system were already outlined in Mazor and Laurent (2005). Here a principal component analysis on the firing rate of a population of PNs revealed different attractors for different odors. These attractors show two transients and one fixed point, but transients are most significant for an efficient odor classification. This addressed for the first time the importance of transient dynamics to explain and understand neural coding and information processing in the MBs. Following these results, we hypothesized that the role of transient dynamics is relevant for the sensory information coding extending the results obtained in locust olfactory system to the fruit fly. This hypothesis well match with the organization properties of the MBs discussed in Nowotny et al. (2003, 2005). Their model is based on spiking neurons and synaptic plasticity, distributed through different layers. The model is able to show consistent recognition and classification of odors. In the study of Nowotny and colleagues, MBs are assumed to be multi-modal integration centers, combining olfactory and visual inputs. As in our current model, the capabilities are independent of the type and the source of information processed in the MBs.

Wessnitzer and co-authors investigated the interaction between MBs and antennal lobes (ALs) and proposed a computational model for non-elemental learning (Wessnitzer et al., 2012). Different levels of learning and reinforcement mechanisms were considered at the stage of the KCs to create a coincidence detector and non-elemental learning. Reward mechanisms are commonly considered for the creation of aversive and appetitive olfactory memories (Schwaerzel et al., 2003) and the role of dopamine is relevant in *Drosophila* (Waddell, 2013). We here extended this scheme to memorize specific parameters involved in the motor-skill learning process. On the basis of fruit fly brain structures and on hypotheses related to information processing and learning mechanisms MBs are a structure able to adapt and memorize relevant parameters involved in motor learning. This improves the fly's capabilities when it is trained in repeating a task like climbing over a chasm. Therefore, a simplified computational model of the MB neuropile was developed using a pool of spiking neurons representing the so-called Kenyon Cells (KCs).

The computational model proposed in this paper for motor learning takes the biological characteristics of the MBs into account and, on the basis of the previously introduced hypotheses, arrives at a neuro-computational structure similar to a Liquid State Machine (LSM) proposed by Maass et al. (2002). The information embedded in the dynamical neural lattice is transferred to the lower motor layers by extrinsic MB neurons that have been modeled as read-out maps.

One fundamental difference between the proposed model and the LSM is the presence of local connectivity among the neurons within the liquid layer. This element of our model deserves particular attention: in fact, the structure configures as a locally connected recurrent neural network which is fairly similar to the Cellular Neural/Nonlinear Network (CNN) structure (Manganaro et al., 1999), a paradigm already used for the generation of complex dynamics and for controlling artificial locomotion (Arena et al., 1999) and perception phenomena (Arena et al., 2009). The other important characteristics of the proposed model is related to the hardware implementation: in fact there are a number of analog/logic VLSI CNN-based chips available which implement digitally programmable analog computers characterized by high computational speed and analog, parallel computation capability, typically used for high frame rate visual microprocessors (Rodríguez-Vázquez et al., 2008). However, the suitable adaptation of the MB structure modeled in this paper as a CNN architecture via the suitable addition of trainable read-out maps would allow for the possibility to adopt a well-assessed reference hardware for the real time implementation of the proposed approach.

From the modeling perspective, the developed structure links two main ideas: high parallelism in brain processing and Neural Reuse (Anderson, 2010). According to the first-mentioned, sensory pathways run in parallel and concur to form abstract schemes of the environmental state, useful for motor actions or abstract decisions. The Neural Reuse approach, on the other hand, states that the same neural structure can be concurrently exploited for different tasks. The insect MBs were already addressed as centers where such characteristics could be found, and the control structure herewith introduced makes a step forward to derive an efficient computational model directly useful as a robot behavioral controller (Arena and Patané, 2014).

## 2. Motor-skill learning in insects

Among the different forms of neural adaptation encountered in animals, motor-skill learning is a fundamental capability needed to survive in dynamically changing environments and also to cope with accidental impairments of animal's limbs.

Motor-skill learning can be defined as the process to acquire precise, coordinated movements needed to fulfill a task. Due to the importance of this capability, sensory-motor conditioning was one of the earliest types of associative learning found in cockroaches and locusts. It has been demonstrated in the ventral nerve cord of insects (Horridge, 1962) and is probably ubiquitous in moving animals (Byrne, 2008; Dayan and Cohen, 2011).

The motor-skill learning system incrementally improves the motor responses by monitoring the resulting performance: this process guides the adaptive changes. By exploiting the involved sensory motor loops, agents apply operant strategies during motor learning: when a movement is performed, sensory feedback is used to evaluate its accuracy (Brembs and Heisenberg, 2000; Broussard and Karrardjian, 2004).

In insects there are different examples of motor learning processes that adapt motor schemes to specific tasks. For instance, honeybees can adapt the antennal movements to an obstacle after a prolonged presentation of this obstacle. Furthermore, the use of an outside rewarding mechanism dramatically speeds-up the learning process (Erber et al., 1997).

Other insect behaviors involving motor learning were reported by Mohl (1993); he investigated the relevance of proprioception during flight in locust. In an interesting paper on *Drosophila* motor-skill learning capabilities (Wolf et al., 1992), a series of conditions has been identified for proper motor-skill learning.

First, the fly has a desired target to reach; to fulfill this aim, a number of motor programs are activated in a random sequence. Efference copies of the motor programs are compared with references and if, for a given motor behavior, a meaningful correlation is found, this is applied. Other studies in this direction were performed on bumblebees (Chittka, 1998) and butterflies (Lewis, 1986).

Behavioral studies on insects confirmed that they are able to show sophisticated and adaptive motor-control strategies requiring the joint coordinated activity among the limbs. A particularly suitable experimental setup to inspect motor learning capabilities is the behavioral paradigm of gap crossing, first described by Blasing and Cruse (2004) and Blasing (2006) in relation to stick insects, by Pick and Strauss (2005) for *Drosophila* and in Goldschmidt et al. (2014) where the coackroach capabilities were considered. Flies with a body length of typically 2.5 mm (and with their wings clipped to disable flight) can cross gaps of up to 4.3 mm when fully exploiting their biomechanical limits. Direct observation and high-speed video analysis of the gap climbing procedure (see Pick and Strauss, 2005 and videos supplied) outlined that flies first visually estimate the gap width via parallax motion generated while approaching the gap. Then, if they consider the gap as being surmountable, they initiate the climbing procedure by combining and successively improving, through several attempts, a number of parameters for climbing. The hind legs are placed as close as possible near the proximal edge; the middle legs are attached to the proximal side wall of the gap and arrange the body horizontally; the front legs stretch out to attach to the opposite gap side. Then the middle legs are detached from the proximal side, swing over and are attached to the distal side surface of the gap. Finally, the hind legs are detached and the fly moves toward the other side. These experiments clearly show that several parameters are modified from their nominal values (for normal walking) and also combined together in several successive phases to maximize the climbing performance.

Later it was shown by Kienitz (2010) that flies improve their climbing abilities when they iteratively climb over gaps of the same width. The short-term improvements after 24 training trials within 1 min were seen in tests 20 min after training; they are missing in plasticity mutants. Rescue of plasticity in the MBs was sufficient to restore the motor-learning capacity. The finding that plasticity in MBs is a prerequisite for motor learning will be taken as our working hypothesis for the development of the proposed computational model. Experiments on gap crossing were also performed with stick insects (Blasing and Cruse, 2004; Blasing, 2006). In these works the authors outlined the role of single leg movements, searching reflexes, and coordination mechanisms as important to fulfill the task. A model of gap crossing behavior was implemented extending a previously developed bio-inspired network Walknet (Cruse et al., 1998), to reach simulated results comparable with the biological experiments. Here the gap crossing issue was considered as an extension of normal walking behavior with only limited modifications. In our work we reached a similar conclusion though starting from quite different models. In fact the CPG for normal walking is maintained whereas only a parameter adaptation was introduced to efficiently implement climbing.

The climbing capabilities of other insects like cockroaches were also considered to develop experiments on obstacle climbing and gap crossing using hexapod robots (Goldschmidt et al., 2014). The presence of an actuated joint in the robot body was exploited to improve the capabilities of the system to face with complex situations including gaps and obstacles (Goldschmidt et al., 2014; Dasgupta et al., 2015). In Pavone et al. (2006), the sprawled posture was a key element for solving the obstacle-climbing issue. In other cases the presence of spoked legs is a simple and efficient solution to improve power efficiency and walking capabilities in presence of obstacles (Moore et al., 2002). In some cases hybrid legged and wheeled robots try to take the advantages of both solutions (Arena et al., 2010).

Whereas, these approaches exploit the mechanical structure, other strategies instead consider primarily the adaptive capabilities of the control structure. For instance, for solving the antenna motor control problem, in Krause et al. (2009) an echo-state network was applied to generate the antenna movements in a simulated stick insect robot. The network was able to store specific trajectories and to reproduce them creating smooth transitions between the different solutions available, depending on the control input provided.

Distributed recurrent neural networks, working as reservoirs, were also used in Dasgupta et al. (2015) to create a forward model needed to estimate the ground contact event in each leg of a walking hexapod robot. The prediction error has been used to improve the robot walking capabilities for different types of terrains.

Our approach belongs to this last type of strategies, since it takes into account primarily the adaptive capabilities of a recurrent spiking network to solve a specific motor learning issue.

In fact, in our work, we considered only obstacle climbing scenarios because our *Drosophila*-like hexapod robot does not contain body joints (i.e., as exploited in Dasgupta et al., 2015 to facilitate also gap crossing); on the other hand it is unfeasible to include in the robot the adhesive capabilities of fly leg tips. Moreover, we assumed that the same computational structure as that one involving the MBs for gap climbing tasks is also involved in obstacle climbing. In the proposed example the external information used to characterize the scenario to be faced, was reduced to the obstacle height (e.g., acquired through a simple visual processing method) in order to learn the set of parameters that allow to fulfill the climbing task.

In particular, the MB intrinsic neurons are here modeled as a spiking network working as a reservoir, able to generate a rich, input-driven dynamics that is projected to other neural centers using read-out maps that work as MB extrinsic neurons. An important added value obtained through the learning process consists in allowing a generalization of the learned data: in fact the network can generate the suitable output signals also for input patterns not included in the learning set by interpolating the memorized functions.

## 3. Modeling motor-skill learning

### 3.1. Known and hypothesized biological functions

Tasks related to motor-skill learning need a specialization of motor functions to optimize performance.To fulfill this aim, a strategy for searching for the most suitable system parameters to be applied for modulating the leg trajectories is envisaged. The generation of pseudo-random parameters constrained only by the insect's body parameters is the initial step needed to improve the ongoing solution iteratively by trial and error. The searching process will produce a subset of successful attempts used to improve the overall system performance, storing the new set of suitable parameters evaluated on the basis of an internal reward function.

In insects, thoracic ganglia can be in charge for the generation of these trials (Horridge, 1962), but MBs should mediate the selection process consisting in a statistical shaping and in the final choice of the successful parameters that modulate the basic behaviors (Kienitz, 2010). Such learning processes are the basic ingredients for the implementation of a short-term working memory.

**Figure 1:**
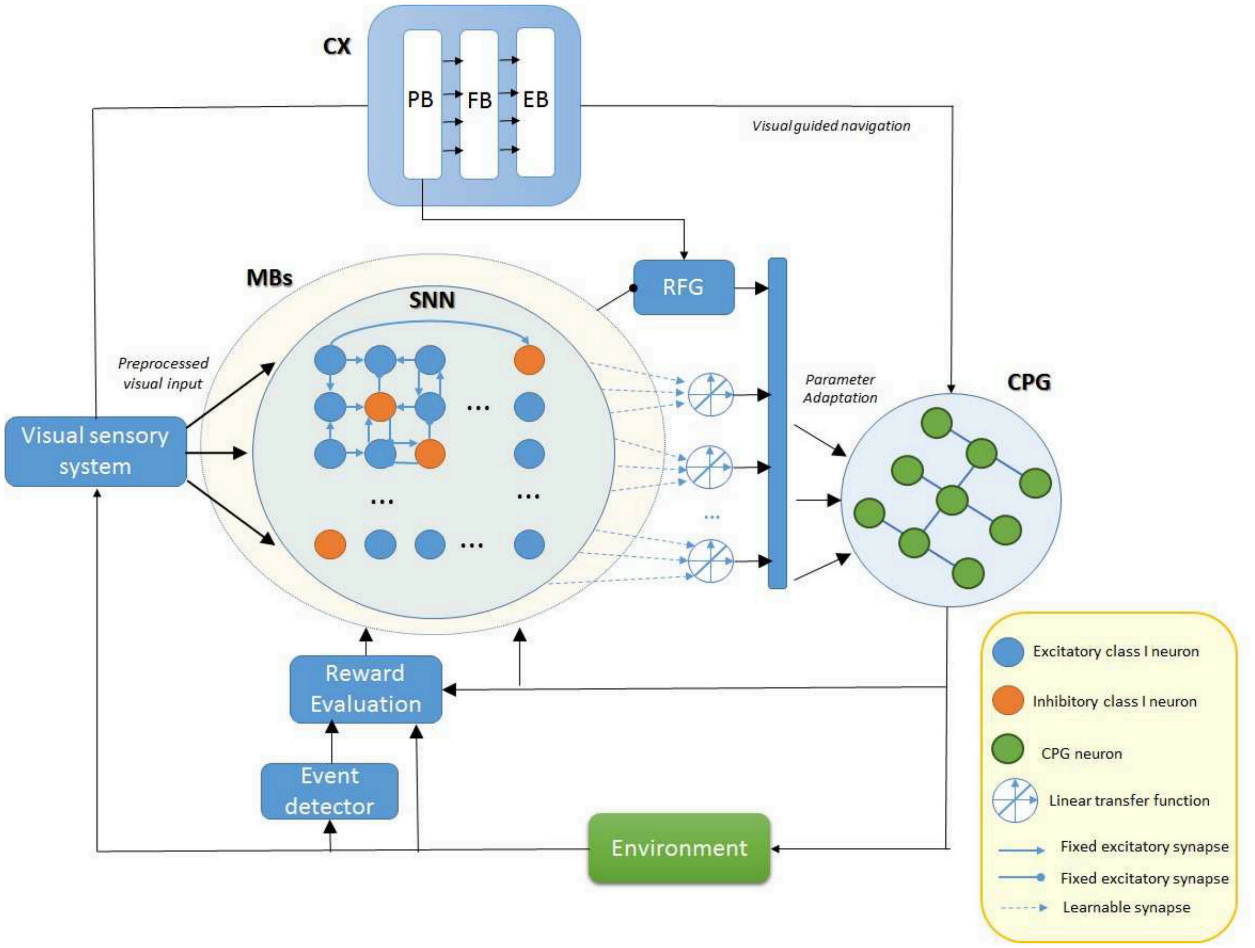
**Block diagram illustrating the role of different fly brain neuropils involved in motor control**. Our model assumes that the parameter adaptation for the modulation of the ongoing behavior is performed by the MBs that receive reinforcement signals in form of dopaminargic/octopaminergic neuron activity and elaborate the learning process using a spiking neural network (SNN). The robot performs a visually guided navigation that acts on the Central Pattern Generator (CPG) structure to control locomotion. A reinforcement signal is generated for the MBs whereas a Random Function Generator (RFG) is used to include fluctuations in the set of control parameters for the CPG. The SNN function is used to memorize the temporal evolution of the modulated parameters if this improves the final motor behavior during learning.

A neuro-control block scheme model is shown in Figure 1 where the main elements involved in the proposed model of motor-skill learning are depicted. Plasticity and learning is ubiquitous in the model due to the complexity of the brain functions but for the aim of the proposed work we focused our attention only on specific parts. Therefore, we considered all the interconnections to be fixed except the synaptic output of the MBs, as will be discussed in details in Sections 3.2 and 3.3, in relation to the motor system (CPG). Plasticity and learning inside other blocks, including the visual sensory and pre-processing system, are not treated in this work.

The central complex (CX) is an excitatory center responsible for behavior activations on the basis of visual and mechanosensory inputs. The input signals are here processed through a series of substructures: the protocerebral bridge (PB), the fan-shaped body (FB), and the ellipsoid body (EB) (Hanesch et al., 1989; Strauss, 2002). Moreover, the PB is directly involved in motor control; it is also responsible for the stabilization of the walking direction (Triphan et al., 2010). On the contrary the MBs seem have an inhibitory effect and are fundamental for the adaptive termination of behaviors (Mronz and Strauss, 2001).

MBs present a large complexity at the level of the calyx, due to the different KC types and their interconnection. From the modeling point of view, KC types could be implemented through different non-linear functions (or dynamical systems). No information is available on the dynamics of these neurons and electrophysiological data are in short supply. On the other side powerful neurogenetic tools are available for the fruit fly which allow for precise manipulations of the nervous system in order to address links among specific neural substrates, their functions and specific behaviors they are responsible for.

Learning in *Drosophila melanogaster* has revealed multiple memory types and phases and recent investigations underlined that not all memory processes occur in MB neurons (Wu et al., 2007; Zhang et al., 2013).

Here we hypothesize that the CX and in particular the PB plays a role in motor learning: it performs adaptation of the motor system parameters shaping the motor behavior while the insect performs a task. The involvement is plausible as the PB seems to control step length for direction (Strauss, 2002; Triphan et al., 2010). This variability is attained in our model (see Figure 1) through a random function generator (RFG) which perturbs some relevant leg control parameters. This strategy generates perturbed leg trajectories. On the basis of the expected results, the on-going behavior is evaluated and eventually, MBs receive a reinforcement signal via extrinsic dopaminergic and octopaminergic neurons (Schwaerzel et al., 2003). Memory consolidation occurs overnight. After consolidation, the MBs are assumed to inhibit the perturbation provided by the RFG to allow the memory retrieval. The overall control system designed and implemented, as outlined in the following constitutes a clear example of a bio-inspired embodied, closed-loop neural controller.

### 3.2. MB model for motor learning: working hypotheses

In order to design both a biologically plausible and a computationally feasible model of the MBs, the two following hypotheses were formulated:

- It is possible that different KCs accept different sensorial inputs at the level of the calyx. This assumption regarding different sensory modalities is made in parallel to olfactory learning (Lin et al., 2013).
- Signal processing within the network takes place at two different levels: within the KCs we have a spiking dynamics within locally, randomly connected neurons, whereas, at the level of extrinsic neurons, we have an external learning needed to learn different tasks. This is a working hypothesis, useful, from the one hand, to computationally simplify the model, and, from the other hand, to allow the concept of Neural Reuse to be directly implemented.

The following structural elements can be outlined:

1. Presence of randomly distributed internal connections.
2. Structural and functional correspondence between internal weights, mirroring the connections within the KC lattice, and the output weights, standing for connections among MBs and extrinsic neurons.
3. Possibility of using the same neural lattice concurrently in completely different tasks, following the Neural Reuse paradigm, by separately training different sets of read-out weights. The same network can therefore model a multimodal (and multifunctional) structure, as are the MBs (Arena et al., 2013c). We are hypothesizing that different sets of extrinsic neurons are devoted to map different tasks.

The proposed control scheme has been implemented in a computational model embedded on a robot simulated in a realistic dynamical environment. Referring to Figure 1, the robot navigates driven by vision: the heading commands are provided to the locomotion controller through external stimuli. An evaluation procedure assesses the suitability of the performed actions in solving the assigned task. An event detector triggers the evaluation process.

The reinforcement signal is passed to the MBs to evaluate the changes generated by the RFG and used to update a set of motor control parameters. Successful parameter updates, leading to significant improvements in the climbing behavior lead to memory formation. A SNN was considered as a plausible model to generate the long-term memory of the best parameters selected during the learning process and to guarantee interpolation capabilities important for the generation of feasible behaviors in situations similar to those ones encountered during the learning procedure. Finally a selector block determines if either a random trial can be performed or the information stored in the SNN can be used for the motor actions. Among the different kinds of neural networks used for solving problems like navigation (Tani, 1996), multi-link system control (Cruse, 2002) and classification, a lot of interest was devoted to Reservoir computing, which mainly includes two different approaches: Echo State Network (ESN) and LSM (Jaeger, 2001; Maass et al., 2002). In previous studies the idea to use non-spiking Recurrent Neural Networks to model the MBs memory and learning functions was explored (Arena et al., 2013a). The core of the newly proposed architecture, inspired by the biology of MBs', resembles the LSM architecture. It consists of a large collection of neurons, the so called liquid layer, receiving time-varying inputs from external sources as well as recurrent connections from other nodes in the liquid layer. The recurrent structure of the network turns the time-dependent input into spatio-temporal pattern in the neurons. These patterns are read out by linear discriminant units. In the last years LSM are becoming a reference point in replicating brain functionalities. However, there is no guaranteed way to analyze the role of each single neuron activity on the overall network dynamics: the control over the process is very weak. This apparent drawback is a consequence of the richness of the dynamics potentially generated within the liquid layer. The side advantage is that the high dimensional complexity can be concurrently exploited through several projections (the read-out maps) to obtain non-linear mappings useful for performing different tasks at the same time. The proposed network differs from the structure reported in Arena et al. (2013a) in many aspects: it consists of a lattice of inhibitory and excitatory spiking (instead of non-spiking) neurons with a random connectivity, which is mainly local (instead of non-local). Moreover, the network configuration in Arena et al. (2013a), for solving the motor learning problem, required a much larger network configuration. This could be addressed to the much richer dynamics generated within the SNN (see Section 5.1). Inputs are here provided as currents that, through a sparse connection, reach the hidden lattice (i.e., the liquid layer). Multiple read-out maps, fully connected with the hidden lattice, can be learned considering the error between the network output, collected through an output neuron for each read-out map, and the target signal. The network details are illustrated in the next section.

### 3.3. Network structure and parameters

Following the biological hints, proposed hypotheses and suggestions from the classical LSM paradigm, the MBs' structure involved in motor learning has been modeled as a spiking-based network consisting of three layers: an input layer, a hidden recurrent neural lattice, and an output layer. The input layer behaves like a filter that randomly redirects input stimuli to a reduced number of neurons in the hidden-layer (KCs lattice). The connectivity percentage used in this work is 15% from the input layer to the KC layer.

The hidden layer is a SNN (i.e., the reservoir network), where each unit is an Izhikevich Class I spiking neuron (Izhikevich, 2000) organized in a square topology with toroidal boundary connections. The regular distribution of the neurons in a square-shaped lattice was selected because, for computational reasons, we considered the simplest structure where we can perform distance metrics. The following differential equations describe the model:

(1) $$\begin{array}{l} \dot{v}=0.04v^{2}+5v+140-u+I\\ \dot{u}=0.02(-0.1v-u) \end{array}$$

following spike-resetting condition:

(2) $$\text{i}\text{f}\;v\geq 0.03,\;\text{t}\text{h}\text{e}\text{n}\{\begin{array}{c} v\leftarrow -0.055\\ u\leftarrow u+6 \end{array}$$

Here *v* is the membrane potential, *I* is the synaptic current and *u* is a recovery variable. Izhikevich neural models are well-known in literature for offering a good compromise between biological plausibility and computational efficiency.

Neurons are connected through synapses: here the spike-rate from the pre-synaptic neuron is transformed into a current for the post-synaptic one. The response of the synapses to a pre-synaptic spike is as follows:

(3) $$\begin{array}{l} \varepsilon (t)=\{\begin{array}{c} Wt/\tau \exp(t/\tau ),\;\text{i}\text{f}\;t>0\\ 0,\;\text{i}\text{f}\;t<0 \end{array} \end{array}$$

where τ is the time constant, *t* is the time passed since the last spike arrived at the pre-synapse and *W* is the synaptic efficiency. This last parameter can be modulated by learning. This synaptic model was also used to connect the lattice neurons to the output neurons.

The fraction of inhibitory neurons in the pool is about 10%. The connections within the lattice are represented by a synaptic weight with a random uniform distribution in the range (0.5–1.5), the input weights are equal to 1. The weights of the read-out map are subject to training. The generation of the inter-layer synaptic connectivity depends on a probabilistic function of the distance *d*_*i, j*_ between the presynaptic (*i*) and postsynaptic (*j*) neurons:

(4) $$P_{ij}=k*C_{i,j}$$

where

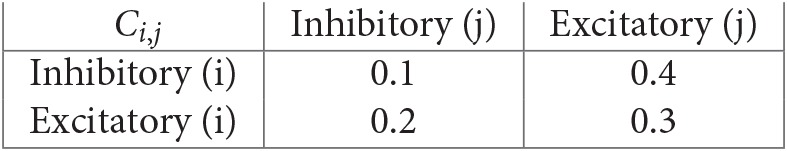



and

(5) $$\begin{array}{c} k=2\;\text{i}\text{f}\;d_{i,j}\leq 1\\ k=1\;\text{i}\text{f}\;1<\;d_{i,j}\leq 2\\ k=0\;\text{i}\text{f}\;d_{i,j}>2 \end{array}$$

The parameters *C*_*i, j*_, reported in the previous table, have been chosen according to Maass et al. (2002). The distance *d*_*i, j*_ = 1 is calculated, either for horizontal or vertical adjacent neurons, considering the neurons as distributed on a regular grid possessing toroidal boundary conditions. From the relations above it derives that the connectivity realized within the lattice is local; this is an important element that facilitates a potential hardware implementation of the control system where the number of connections is drastically reduced and limited to each neuron neighborhood.

The time constant in Equation (3) was randomly chosen among the values τ = 5, 10, 30, and 50 ms. This variability improves the dynamics potentially shown by the network as will be discussed in the following sections. The values of the synaptic time constant have been chosen to obtain significant dynamics in the simulation time window that is limited to 150 ms.

The output layer consists of a series of output neurons, modeled with a linear transfer function and fully connected with the hidden lattice. The output weights are randomly initialized in the interval (−1, 1) and are subject to learning. The integration step used for the reported simulations was fixed to *dt* = 1.5 ms.

### 3.4. Learning mechanism

The time evolution of the target signals that the network need to memorize is generated by shaping the lattice dynamics using read-out maps. An incremental learning rule based on the Least mean square algorithm is adopted to update the synaptic weights of each read-out map. The learning process, resembling the classical delta rule, depends on the lattice activity and on the error between the current output and the desired target. The updating rule of the synaptic weights is here reported:

(6) $$W_{i,j}(t+\delta t)=W_{i,j}(t)+\eta *Z_{i,j}(t)*E(t)$$

where η is the learning rate, *Z*_*i, j*_(*t*) is the synaptic output of the neuron (*i, j*) at time *t* and *E*(*t*) is the error between the network output neuron and the desired target. Another possibility consists of cumulating the weight variations during the simulation time window, to finally apply the cumulative result during the last simulation step.

## 4. Simulation results

**Figure 2:**
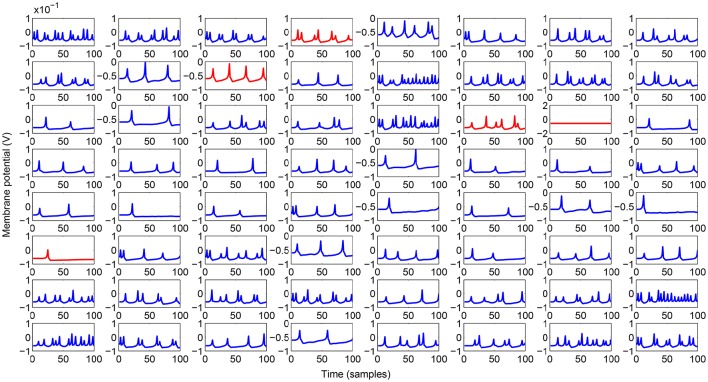
**Spiking activity of the lattice while generating the output signal during the testing phase**. Inhibitory neurons are outlined in red.

**Figure 3:**
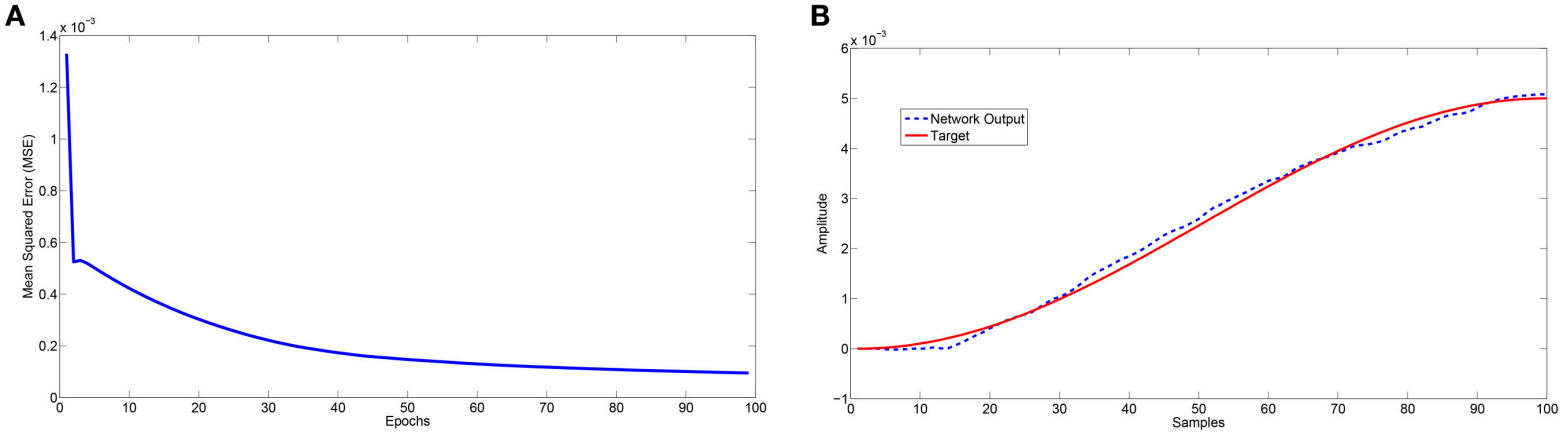
**(A)** Trend of the mean square error during 100 learning trials (epochs), **(B)** Comparison between the expected output and the network approximation at the end of the 100 learning trials.

The analysed motor learning process consists of adopting a series of perturbations on specific leg control parameters to reach a success in the assigned task. To apply a smooth perturbation, we adopted as target signal, a cosinusoidal function, whose final value corresponds to the parameter to be applied. In the following simulations we adopted a lattice with 8 x 8 neurons that is a good compromise to obtain a considerable variety of internal dynamics. The learning process needs a series of iterations (here called epochs) to successfully store the information in the read-out maps. In the following analysis we considered 100 epochs with a learning rate η = 0.5. During each epoch the network is simulated for 100 integration steps. A typical activity of the neural lattice is shown in Figure 2. The input given to the network through an external current is related to the information acquired from the environment and, using the learning rule in Equation (6), we can determine the weights of the read-out map in order to follow a target signal as shown in Figure 3.

**Figure 4:**
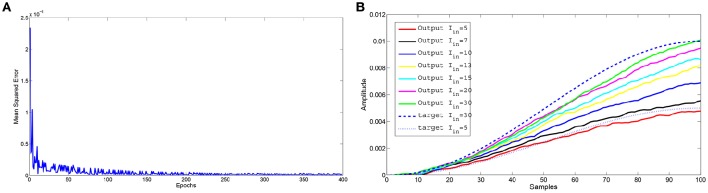
**Learning example with two different input-target pairs: input current 5 and 30μA**. The interpolation capabilities were tested using different inputs: 5, 7, 10, 13, 15, 20, and 30μA. **(A)** Trend of the mean square error during 400 epochs used to learn the two input-target patterns. For each epoch we provided alternatively either one or the other input-target pair, allowing the learning process to update the read-out weights obtained during the previous learning epoch. **(B)** Comparison between the expected output and the network approximation at the end of the epochs.

The network allows to interpolate the information acquired during learning as illustrated in Figure 4. During the 400 epochs used for the learning phase, two distinct output signals, corresponding to different input currents (*I*_*in*_ = 5 and 30μA), were learned. During the testing phase, besides the two inputs already used in the learning phase, also other input currents were provided obtaining plausible behaviors that interpolate the dynamics of the two learned target signals.

**Figure 5:**
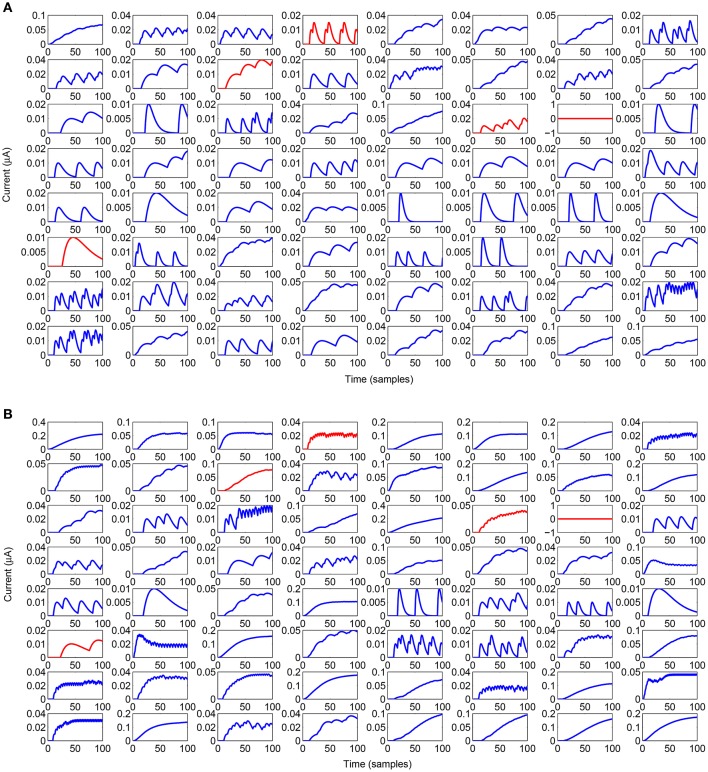
**Behavior of the input currents provided to the output neuron from the lattice when the input layer provides a current of 5μA (A)** and 30μA **(B)**. Inhibitory neurons are outlined in red.

Figure 5 reports the synaptic activity (Equation 3), in the form of currents generated by the lattice before learning, weighted by the read-out map and summed over the 100 samples for all the spikes emitted by the neurons to reproduce the two target signals. It can be noticed that even a lattice with a limited number of neurons can produce a large variety of dynamics that can be combined by the output neurons. The differences in the synaptic time constant, play a role in increasing the richness of dynamics during the network activity. It is also evident how sensitive the structure is to a change in the input current provided to the lattice; it can generate a drastic change in the temporal evolution of the network dynamics. This allows for a high interpolation capability. The use of spiking networks over nonspiking ones to model nonlinear dynamics is often considered as an additional complication. Our case is an example to the contrary. In fact, in Arena et al. (2013a) nonlinear nonspiking recurrent neural networks were used to model MB activity: the non-spiking recurrent configuration, suitable for solving the motor-learning problem was fixed to 140 non locally connected units, whereas the results presented in this paper were obtained via a network with 64 spiking locally connected neurons in the liquid layer.

## 5. Motor learning: application to climbing

### 5.1. Learning new motor activities in a stable locomotion controller

The insect brain can be considered as a parallel computing architecture where reflexive paths serve the basic needs for survival, whereas learned paths allow the formation of more complex behaviors.

**Figure 6:**
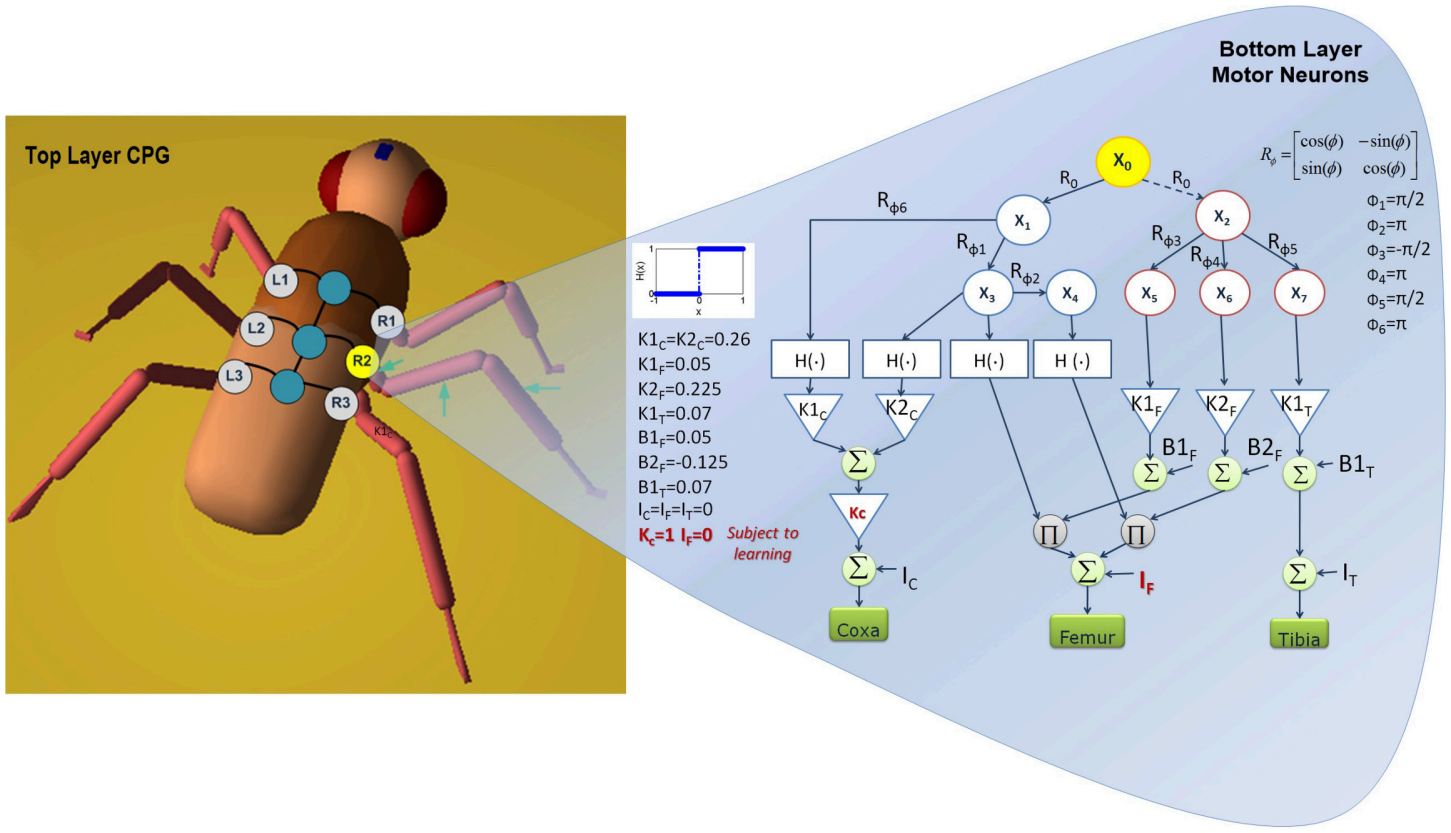
**Neural network scheme: the top layer generates a stable gait pattern, whereas the bottom layer is constituted by additional sub-networks generating the specific reference signals for the leg joints**. The network devoted to control a middle leg is reported. The parameter adapted during the learning process for the middle legs are indicated in red.

Regarding motor activities in insects, the thoracic ganglia are mainly responsible for the generation of locomotion gaits, and the Central Pattern Generator (CPG) has widely been accepted as being the core unit for locomotion control but its fine-tuning is usually achieved by sensory information. The approach proposed here considers the task of motor learning as that of finding a suitable way for modifying the basic motor trajectories on the single leg joints so as to improve motor-skills in the light of novel conditions imposed by the environment. Using a control approach, we can realize motor-skill learning through a hierarchical adaptive controller, where, when facing novel conditions, some parameters controlling the leg joint trajectories are modulated. These modulations, shaped by the kinematic constrains, realize novel leg trajectories which are then applied to assess their suitability for the task. Once the former locomotion conditions are restored, these modulations are withdrawn and the baseline stable locomotor activity re-emerges. Sets of successful parametric values are retained, so that they can be re-applied whenever similar conditions should be encountered again. The locomotion controller is made up of basically two networks: one is devoted to generate a stable phase displacement among the legs; the other is shaped on the specific kinematic structure of each leg and constituted by several motor neuron structures, as illustrated in Figure 6. The basic cell characterizing the CPG architecture is described by the following equations:

(7) $$\{\begin{array}{c} {\dot{x}}_{1,i}=-x_{1,i}+(i+\mu +\varepsilon )y_{1,i}-s_{1}y_{2,i}+i_{1}\\ {\dot{x}}_{2,i}=-x_{2,i}+s_{2}y_{1,i}+(i+\mu -\varepsilon )y_{2,i}+i_{2} \end{array}$$

with *y*_*i*_ = *tanh*(*x*_*i*_) and the parameters for each cell: μ = 0.23, ε = 0, *s*_1_ = *s*_2_ = 1, *i*_1_ = *i*_2_ = 0 generate a stable limit cycle (Arena et al., 2005). μ is chosen to approximate the dynamics to a harmonic oscillation. The CPG network is built connecting adjacent cells using links expressing rotational matrices *R*(ϕ), as follows:

(8) $${\dot{x}}_{i}=f(x_{i},t)+k\sum_{j\neq i}(R(\phi_{i,j})x_{j}-x_{i})\text{ with }i,j=1,\cdots ,n$$

where the summation involves all the neurons *j* which are nearest neighbor to the neuron *i*; *n* is the total number of cells; *f*(*x*_*i*_, *t*) represents the reactive dynamics of the i-th uncoupled neurons as reported in Equation (7) and *k* is the strength of the connections. The sum of terms performs diffusion on adjacent cells and induces phase-locking as a function of rotational matrices (Seo and Slotine, 2007). The presence of local connections is an important added value because it reduces the system complexity in view of a hardware implementation. The bottom layer is designed based on the desired kinematic behavior; it is directly correlated to the morphology of the limb. The network controlling one of the middle legs is sketched in Figure 6. The CPG neuron identified with the label *R*2 is connected through rotational matrices with different angles to a network of motor neurons arranged in a directed tree graph that uses the same neuron model as CPG. The blocks *H*(•) are Heaviside functions and are used to distinguish, within the limit cycle, between the stance and swing phases: this allows to associate suitable modulation parameters to each part of the cycle, depending on the morphology of the leg. The signals are finally merged to generate the position control command for the coxa, femur and tibia joints. A detailed discussion on the CPG structure and behaviors is reported in a previous study (Arena E. et al., 2012).

The overall network stability was theoretically proven exploiting tools from partial contraction theory on a network made of nonlinear oscillators with Laplacian couplings. As demonstrated in previous studies, the network for gait control has a diffusive, undirected tree-graph configuration, which guarantees asymptotic phase stability independently of any imposed locomotion pattern (Arena et al., 2011; Arena E. et al., 2012).

The stable phase-locked oscillations generated in that way are passed on to the motor neural network for each leg, whose particular structure controls leg motion while maintaining the imposed phase among the legs. Upon this stable basic locomotor activity, the motor-learning controller is added, whose role is to find suitable modulation of the single-leg motions to learn proper trajectories in the presence of specific needs. Basic motor activities are so disturbed to find new solutions for the leg motions, thus implementing motor-skill learning.

### 5.2. Climbing experiment

Motor-skill learning in the presented multi-limb system is applied to improve the robot capabilities in solving different tasks involving multiple degrees of freedom; here, in fact a fine tuning of parameters is required to modulate the basic cycling behavior in the different legs of the robot.

Among the possible tasks, in the simulation a step-climbing scenario has been considered in the simulation. In nature, insects are continuously faced with uneven terrains and they adapt their motor responses to accomplish tasks like climbing over surmountable objects. Even flying insects, like *D. melanogaster*, show exquisite climbing skills, since searching for food in the near-field and courtship are achieved during walking. The aim of motor learning in our experiments is to improve the climbing capabilities of a simulated robot through the modulation in time of a group of parameters used in the leg motor layer. This simulated scenario is a realistic alternative to the gap climbing scenario used in the biological experiments (Blasing and Cruse, 2004; Pick and Strauss, 2005; Kienitz, 2010; Triphan et al., 2010). In fact, due to the adhesive capability of the fly legs (possessing pulvilli and claws), gap climbing is an affordable task for the real insect, whereas this is extremely difficult for a *Drosophila*-inspired robot that cannot reach the same dexterity as the biological counterpart. In other hexapod robots the presence of an active body joint, inspired by the cockroach, was exploited to improve the system capabilities in gap climbing tasks (Goldschmidt et al., 2014). In our *Drosophila*-inspired robot, due to the absence of this degree of freedom in the body, we considered obstacle-climbing scenarios, which are a challenging task for legged robots that have to improve their climbing capabilities by learning. For a future direct comparison with biological experiments, the new paradigm lends itself for testing real flies. Moreover, the step climbing scenario can be made more demanding by using slippery surfaces which would reduce the advantage of the animal if compared with the robot.

**Figure 7:**
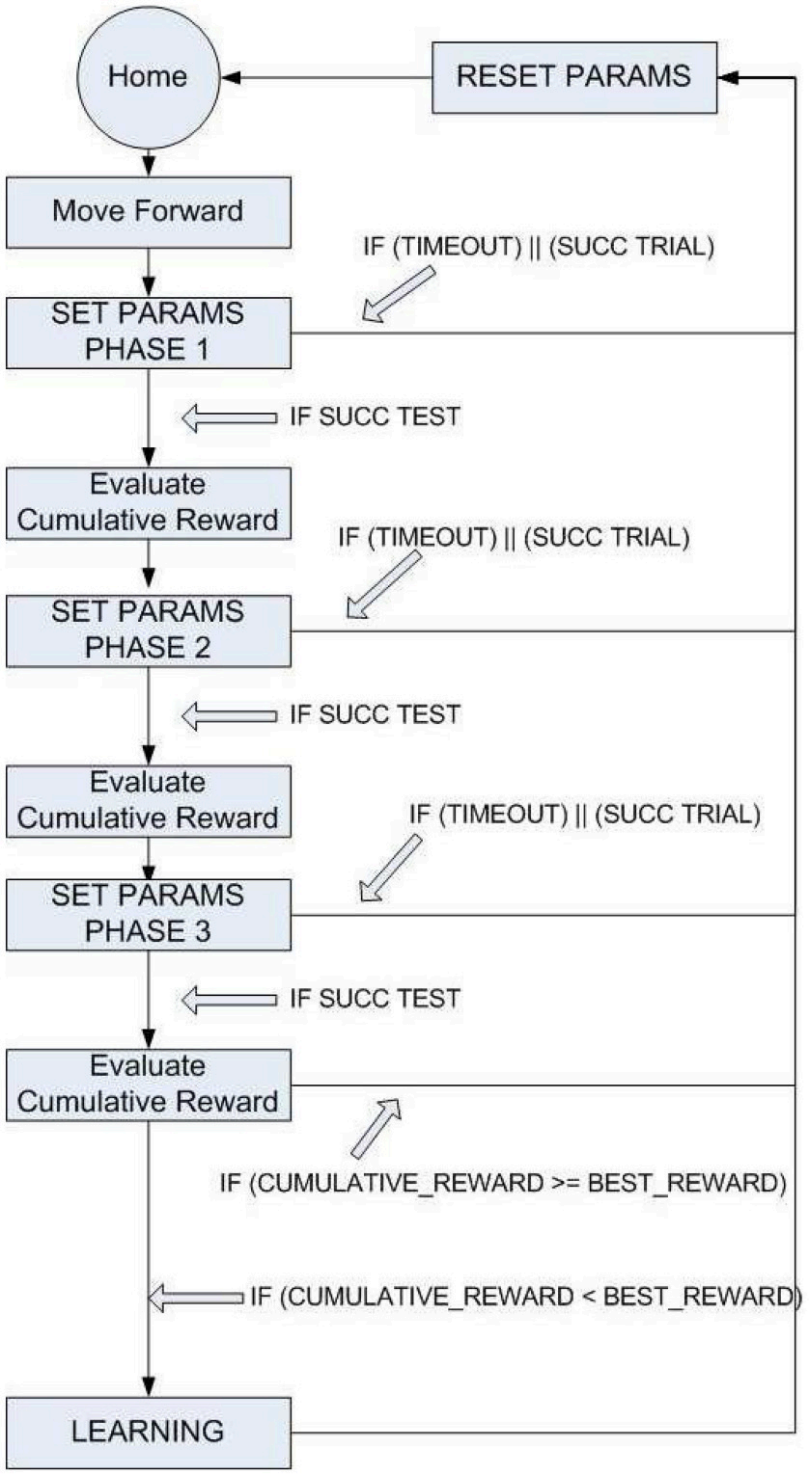
**General scheme of the procedure followed to improve the robot motor-skills in a multi-stages task**. Starting from Home, an event triggers the request of parameter adaptation for the Step 1 that is tried until a success occurs or a time-out is reached. Within the time-out triggered window, it is possible to evaluate the effectiveness of multiple sets of parameters that persist for about a complete cycle of a leg (i.e., overtime). The success is evaluated by a cumulated reward and, if an improvement is obtained, the parameter evolution is stored in the long-term memory (i.e., read-out maps). The other stages follow the same procedure.

Step climbing for a robot is quite a complex task and should involve an optimization method to adapt the joint movements to different surfaces. To simplify the problem, the task was split into different phases shown in Figure 7.

The approaching phase is guided by the visual system that is able to recognize the distance from the obstacle and its height. When the robot's distance from the step is below a threshold, Phase 1 is activated and the parameters of the front legs are adapted using the RFG to modify its movements, in an attempt to find a foot-hold on the step. For sake of simplicity, a subset of parameters available in the adopted CPG was subjected to learning in this phase.

In details, for the coxa joint the bias value, for the femur joint the gain value, for the tibia joint the bias and gain values were selected for learning. This phase leads to a stable positioning of the front legs on the step, with the body lifted off. The extent of the angular motion of the leg joints, caused by the modulation of the parameter profiles, is used as an index of the energy spent in this task and to define a reward function. The reward value is then compared with the previously found best value and, if an improvement is obtained, the new sets of functions are stored in the SNN readout map. For the considered task we have a single lattice with one input (i.e., step height) and a total of ten read-out maps, one for each parameter to be learned for a specific leg joint. The SNN receives as input a normalized value related to the step height and the lattice dynamics generates a spatio-temporal spiking activity that is transformed in a continuous, non spiking signal, through the output synapses that converge on the output neurons, one for each parameter that is subject to learning.

A series of experiments were performed using a step that is insurmountable unless a gait adaptation is introduced: the height of the step is around 0.9 mm, whereby we chose the simulated *Drosophila* body length as 3.2 mm and the average height of the center of mass as to be located at about 1 mm above the ground during forward walking.

**Figure 8:**
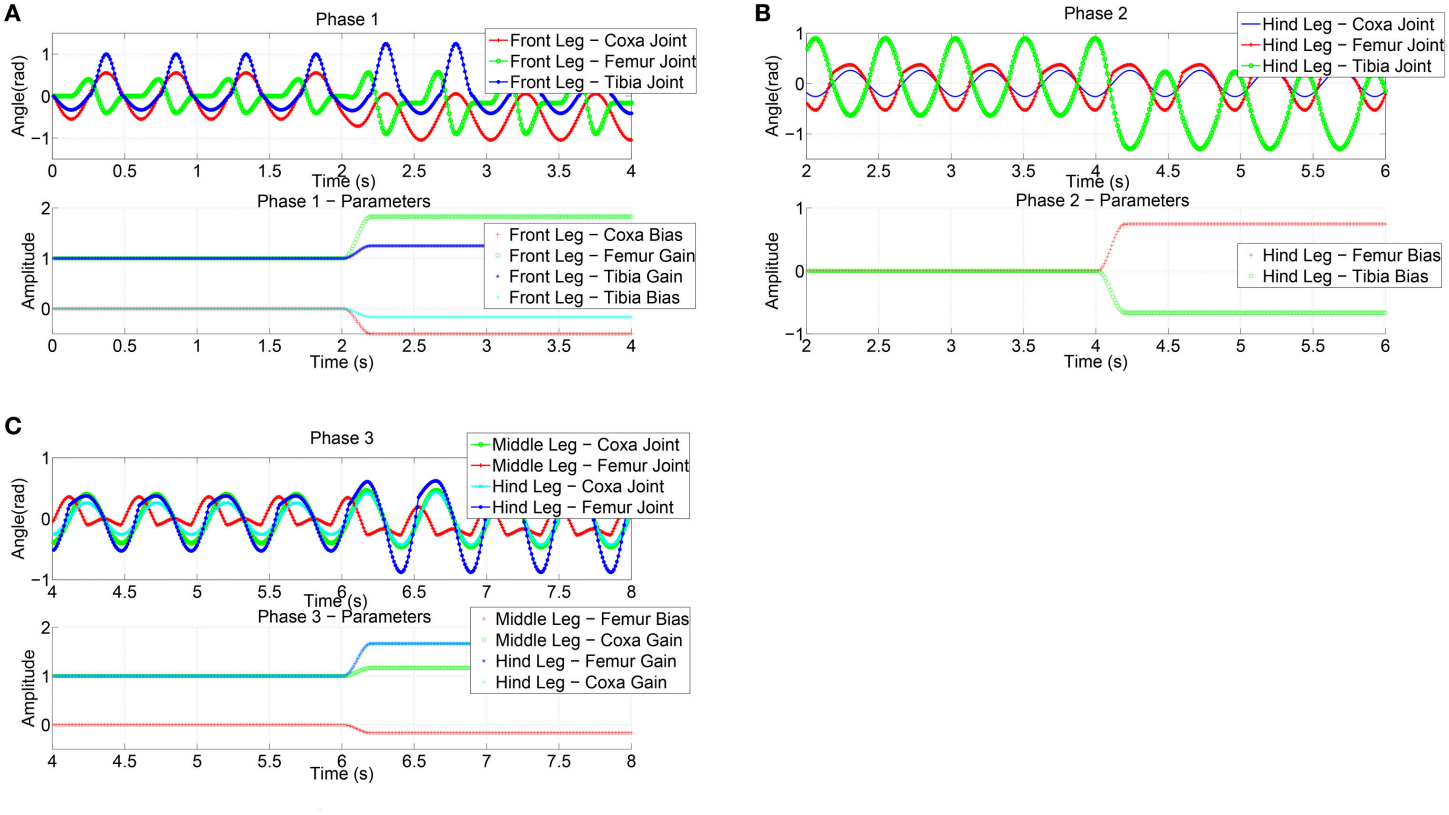
**Effects of the parameter adaptation on the leg joints (only the left body side is shown)**. A limited number of parameters is subject to learning in the three phases of the obstacle climbing procedure: **(A)** in phase 1 only the front legs are involved, **(B)** the hind legs in phase 2, and **(C)** both middle and hind legs in phase 3. The effect of the parameters on the leg joint trajectory is limited to the current phase.

The joint angular positions caused by the parameter adaptation in the anterior legs are shown in Figure 8A. The subsequent phase is similar: here as relevant parameters to be adapted, the bias of femur and tibia joints of the hind legs are considered to facilitate the climbing of the middle legs. The event considered in this phase to evaluate the success and the consequent passage to the successive phase is the horizontal position of the center of mass of the robot with respect to the obstacle. The parameter adaptation results for the second phase are depicted in Figure 8B. During the third phase the robot elevates the hind legs on the step: this is achieved by modulating the gain of the coxa and bias of the femur joint for the middle legs and the gain of the coxa and femur joint of the hind legs (Figure 8C). In the actual experiments the function adopted to deliver the randomly generated parameter modulation on the joints is a cosinusoid, however other functions, like exponentials, quarter sinusoids, or sigmoids could be used. Actually the function reaches the steady state value in a given time window that is a portion of a stepping cycle.

In the dynamic simulation herewith reported, we adopted an integration time dt = 0.01 s, a stepping cycle of about 1.5 s: these conditions, the parameters reach the steady state within [20–60] integration steps. Looking at the learning process, the RFG generates the new parameters to be tested for the first phase. If the trial is successful the robot is re-placed to the starting position to perform a test: this assures the robustness of this new set of parameters. If the robot succeeds, it can proceed to the second phase, otherwise the parameters are discarded and the first phase is repeated. The trial ends when the robot overcomes the last phase or after a given number of attempts (i.e., 15 events). If this time-out occurs, the parameters just used for the phases are discarded because they are not globally suitable for a complete climbing behavior.

**Figure 9:**
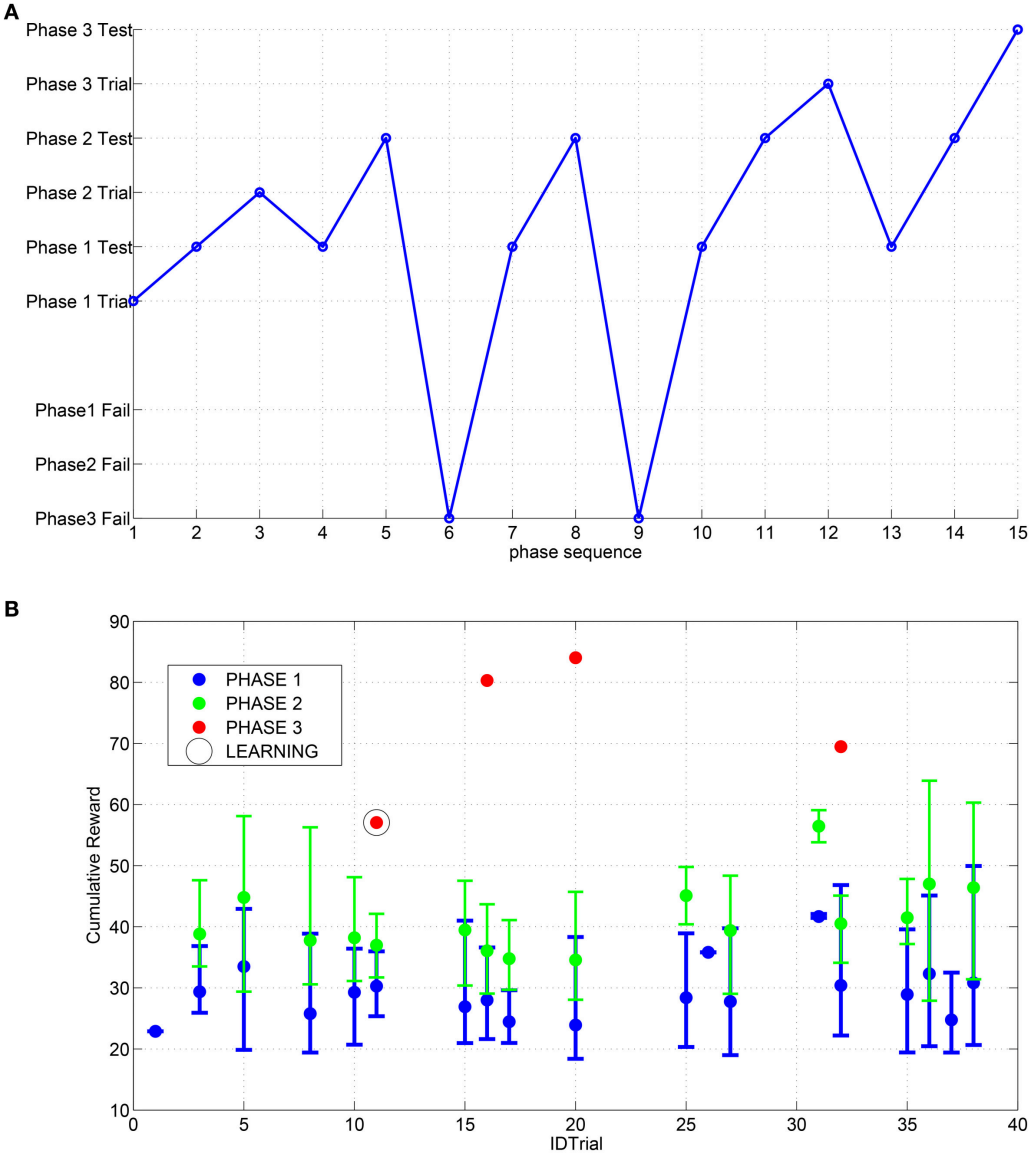
**(A)** Sequence of events obtained during the searching process for the suitable parameters through the RFG. **(B)** Distribution of the cumulative reward for each trial; the error bars indicate the range of excursion between min and max value and the marker corresponds to the mean value. The learning of the SNN is performed only for the IDTrial 11 that is the same as reported in **(A)** because in the other success event for the third step there are no improvements for the cumulative reward.

In Figure 9A an example of a trial is reported: the robot succeeds in the first attempt to find a suitable set of parameters to complete the first and second phase, whereas for the third phase a series of failures both in learning and in test are obtained until the final success is reached (see Supplementary Video 1 for a typical sequence of trials with successes and failures). The success in the trial can be followed by a learning process in the SNN depending on the overall reward value obtained. In Figure 9B the distribution of the cumulative reward in a campaign is shown. For each trial the success condition for each phase can be reached multiple times until the complete climbing behavior is tested successfully or otherwise a time-out occurs. If the obtained cumulative reward (i.e., sum of the rewards for each phase) after the third phase is lower than the previously obtained values, the parameters are learned by the network.

**Figure 10:**
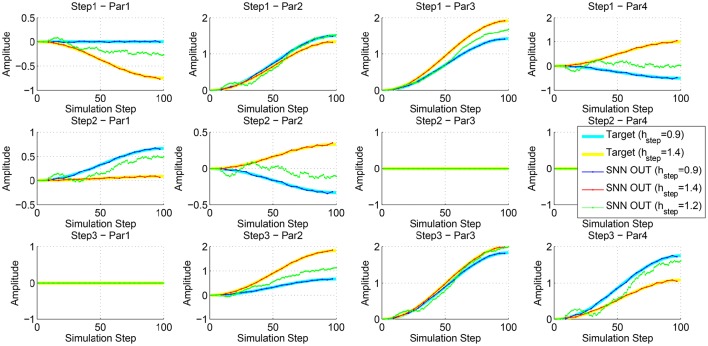
**Comparison between the best parameters provided by the RFG and the output of the SNN after the read-out map learning for a step of 0.9 and 1.4 mm**. Moreover, the output of the network for the input of an intermediate step height is shown.

**Figure 11:**
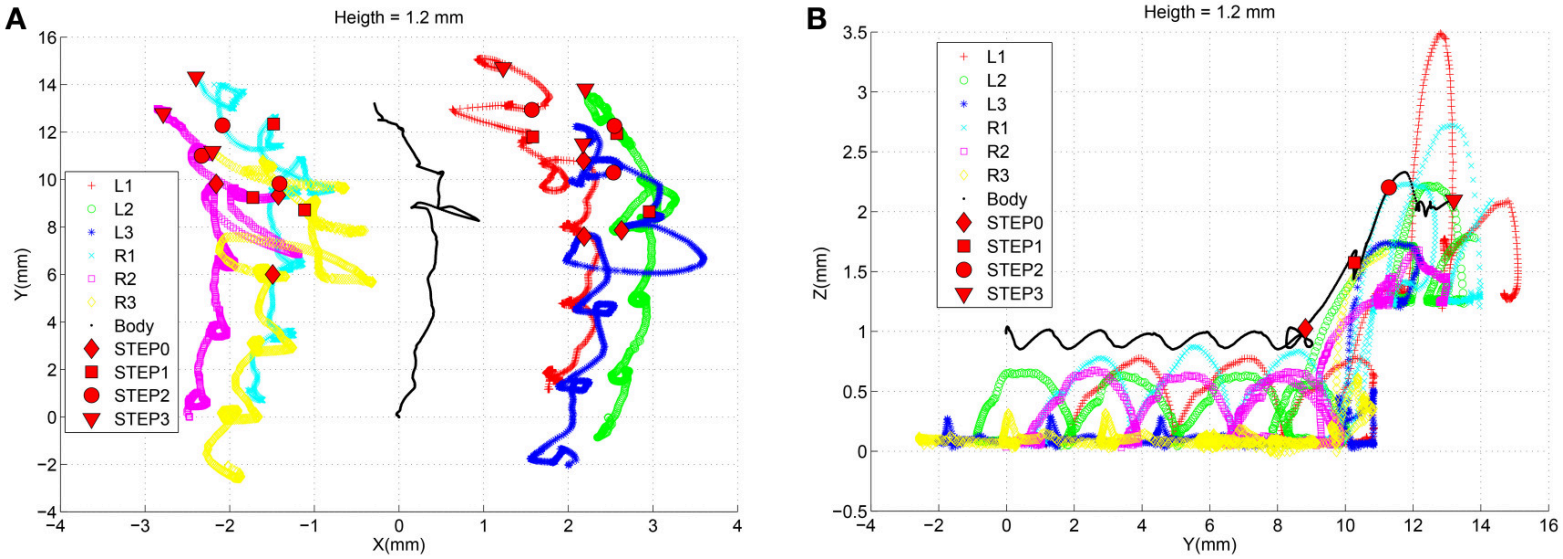
**Trajectories followed by the center of mass of the robot and by the tip of each leg during the climbing behavior facing an obstacle of 1.2 mm**. A marker is placed in the signals to indicate when the robot completes each phase.

**Figure 12:**
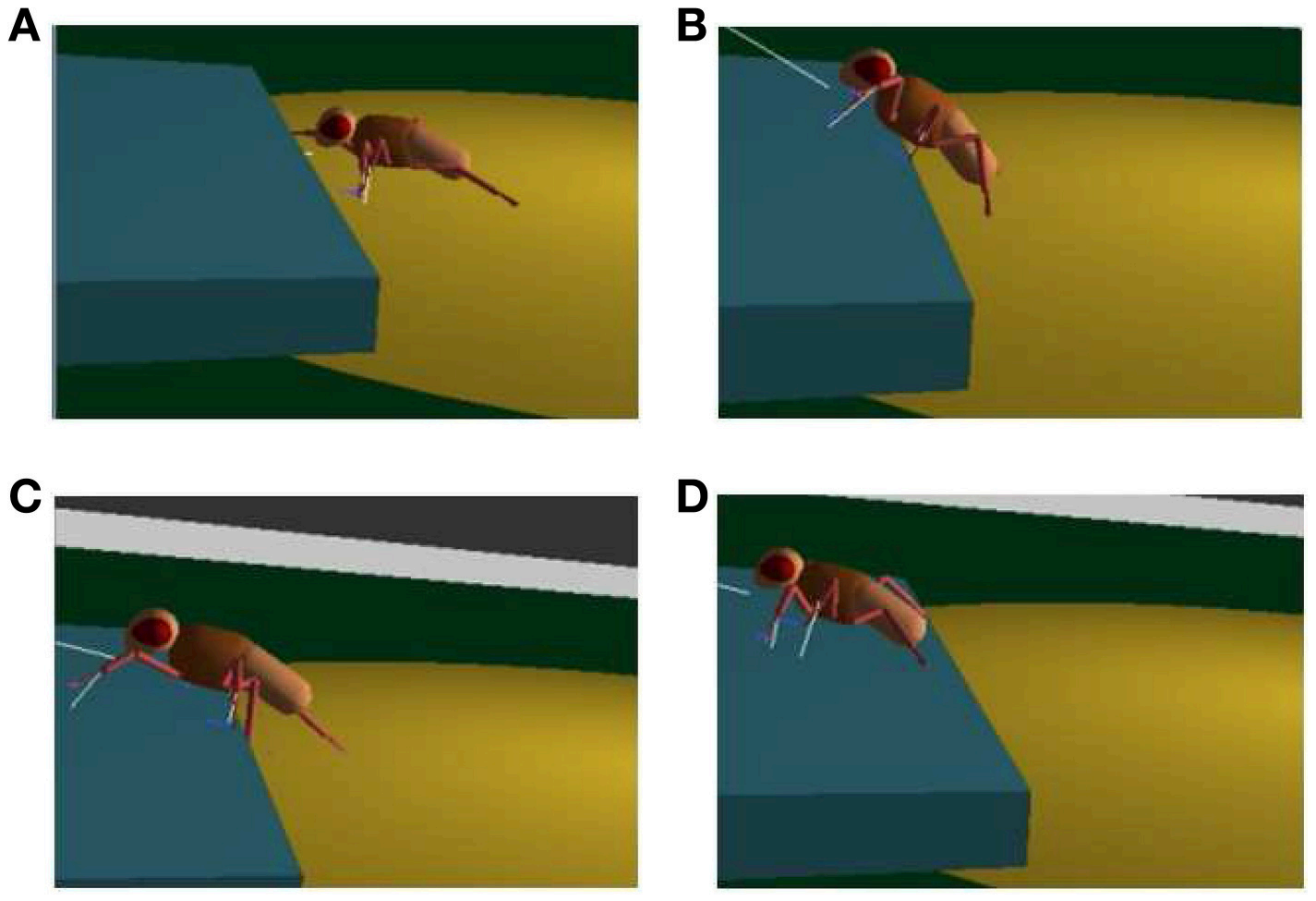
**Snapshots of the fly-inspired robot while climbing an obstacle: the robot approaches the obstacle (A)**; it reaches the step with its front legs **(B)**, middle legs **(C)**, and finally with the hind legs **(D)**.

To evaluate the interpolation capabilities of the network we also performed a series of learning sessions with higher obstacles (i.e., 1.4 mm) and subsequently we tested the robot with a step height never provided during learning (i.e., 1.2 mm). The best-adapted parameters obtained for the two learned step heights are reported in Figure 10 together with the network response to the new step with an intermediate height. The obtained results were tested with the simulated robot obtaining a success in the climbing behavior as reported in Figure 11. This depicts the motion of the robot's center of mass (COM) and of the tips of each leg when climbing a 1.2 mm step. The edge of the step is placed at 11 mm far from the COM home position (along the y axis) (see Supplementary Video 2). Moreover, a series of snapshots outlining the posture of the fly-inspired robot during the climbing task are depicted in Figure 12.

**Figure 13:**
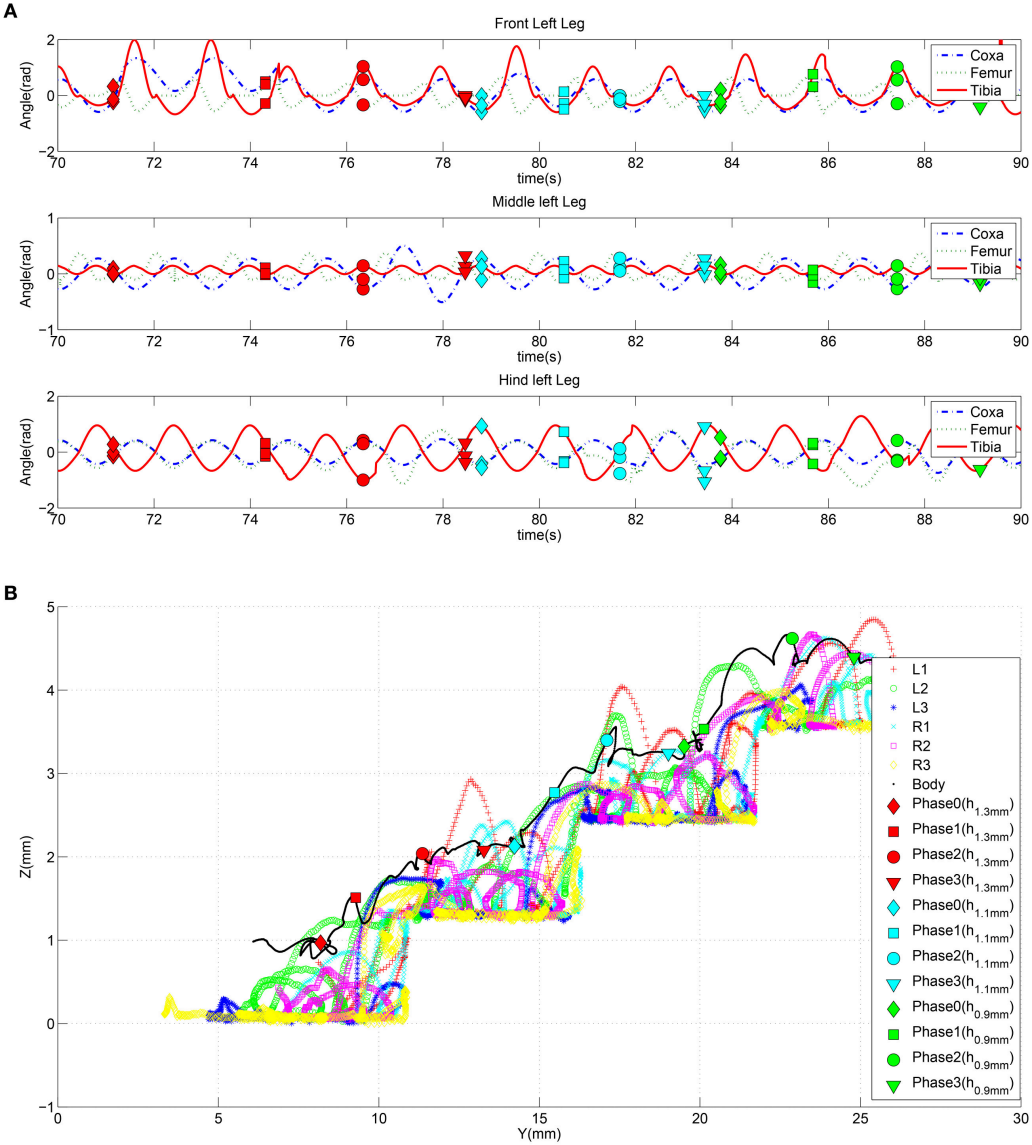
**(A)** Trend of the joint positions for the left side legs when the robot faces a series of obstacles. **(B)** Trajectories followed by the center of mass of the robot and by the tip of each leg during the climbing behavior facing with multiple obstacles with height 1.3, 1.1, and 0.9 mm, respectively. A marker is placed in the signals to indicate when the robot completes each climbing phase.

To evaluate the generalization capability of the control system, the previously learned system was tested in a different scenario where a stair-like obstacle was introduced. The robot followed the same climbing procedure as described above, repetitively applied for the three stair steps encountered on its path with height 1.3, 1.1, and 0.9 mm, respectively. The detection of each obstacle produces an effect at the motor level on the basis of the parameter adaptation mechanism induced by the SNN. Figure 13A shows the trend of the joint position angles for the left-side legs during the whole climbing procedure. The adapted parameters produce changes in the leg movements during the different climbing phases as illustrated in Figure 13 B where the dynamics of the robot COM and the leg tip positions are reported (see Supplementary Video 3).

The results obtained were achieved relying only on the adaptive capabilities of the legs acquired during the learning phase. The body structure was considered rigid as in the fruit fly case. Including in the robotic structure active body joints (Dasgupta et al., 2015), mimicking the body of other insects like cockroaches, would only improve the robot capabilities. Therefore, the proposed control strategy can be also applied to other different robotic structures to improve their motor capabilities in fulfilling either obstacle climbing tasks or other similar scenarios affordable for the robot under consideration.

## 6. Remarks and conclusions

In this paper a bio-inspired, embodied, closed-loop neural controller has been designed and implemented in a simulated hexapod robot that is requested to improve its motor-skills to face unknown environments. Taking inspiration from the insect brain and in particular from the fruit fly, the following hypotheses were formulated: relevant role of MB neuropiles in the motor learning task; direct transfer of the important role of transient dynamics in the olfactory leaning from the locust to the fly brain and further extension to motor learning; design of a neuro-computational model based on a LSM-like structure for the implementation of obstacle climbing in a simulated hexapod robot.

In details, a computational model for motor-skill learning was developed and realized in a dynamic simulation environment. Inspired by behavioral experimental campaigns of motor learning in real insects, the computational structure consisted in a randomly connected SNN that generates a multitude of nonlinear responses after the presentation of time dependent input signals. By linearly combining the output from the lattice neurons with a weighted function, a reward-based strategy allows to learn the desired target by tuning the weights of a readout map. Looking at MBs in insects, the idea of a pool of neurons enrolled to solve different tasks depending on the specific requested output is next to the concept of Neural Reuse which has a number of biological evidences. The reported results demonstrate that the system can learn, through a reward-driven mechanism, the time evolution of several independent parameters related to the leg movements, to improve the robot climbing capabilities when exposed to the step-climbing task. The robot was also able to deal with step heights never presented before, exploiting the interpolation abilities of the proposed network.

## Author contributions

All authors listed, have made substantial, direct and intellectual contribution to the work, and approved it for publication.

### Conflict of interest statement

The authors declare that the research was conducted in the absence of any commercial or financial relationships that could be construed as a potential conflict of interest.
